# Summary of the best evidence for non-pharmacological interventions for dyslipidemia in patients with coronary heart disease

**DOI:** 10.3389/fcvm.2026.1753853

**Published:** 2026-03-11

**Authors:** Zhi Zeng, Xin Zhou, Li Jiang, Yuan Huang, Xiaochun He, Huaili Luo

**Affiliations:** 1 Department of Intensive Care Unit, Mianyang Central Hospital, Affiliated with the School of Medicine, University of Electronic Science and Technology of China, Mianyang, Sichuan, China; 2 Department of Gastroenterology, Mianyang Central Hospital, Affiliated with the School of Medicine, University of Electronic Science and Technology of China, Mianyang, Sichuan, China; 3 Department of Cardiovascular Medicine, Mianyang Central Hospital, Affiliated with the School of Medicine, University of Electronic Science and Technology of China, Mianyang, Sichuan, China

**Keywords:** dyslipidemia, non-pharmacological interventions, patients with coronary heart disease, review, summary of the evidence

## Abstract

**Objective:**

Retrieve, evaluate, and summarize evidence from domestic and international sources regarding non-pharmacological interventions for dyslipidemia in patients with coronary heart disease (CHD), providing reference for clinical practice.

**Methods:**

Following the “6S” evidence pyramid model, a systematic search was conducted for literature on non-pharmacological interventions for dyslipidemia in CHD patients, both domestically and internationally, covering the period from January 2014 to December 2025. Two researchers independently screened and evaluated the studies for inclusion, after which the evidence was synthesized.

**Results:**

A total of 31 studies were ultimately included, comprising 15 English-language publications and 16 Chinese-language publications. Thirty-one evidence points were summarized, covering seven evidence themes: multidisciplinary team formation, assessment, lipid-lowering targets, lifestyle modification, non-pharmacological traditional Chinese medicine (TCM) interventions, health education, and follow-up management.

**Conclusion:**

This study provides a comprehensive summary of the best available evidence on non-pharmacological interventions for dyslipidemia in patients with CHD. The findings can serve as a valuable reference for clinical practitioners, enabling healthcare professionals to develop personalized intervention plans based on the lipid profiles and specific needs of CHD patients.

## 1 Introduction

Coronary heart disease (CHD) is a cardiac condition caused by atherosclerosis of the coronary arteries, leading to narrowing, spasm, or even occlusion of the vessel lumen, which in turn triggers myocardial ischemia, hypoxia, or necrosis (1). It is characterized by high morbidity and mortality rates (2). Research indicates that in developed countries, effective control of risk factors and rational allocation of medical resources have led to a declining trend in CHD prevalence. Conversely, in developing nations, accelerated urbanization and lifestyle changes have driven a rapid increase in its prevalence (3). As a developing nation, China follows the global trend among similar countries, with CHD affecting 11.39 million individuals (4), imposing significant economic burdens on patients’ families and healthcare institutions (5). With an aging population, its incidence and mortality rates are projected to continue rising (6). Dyslipidemia is a major risk factor for CHD recurrence and mortality and is primarily manifested as elevated serum levels of total cholesterol, low-density lipoprotein cholesterol (LDL-C), and triglycerides, coupled with reduced levels of high-density lipoprotein cholesterol (HDL-C) (7, 8). Multiple studies (9–11) demonstrate that effective lipid-lowering therapy not only significantly reduces LDL-C levels in CHD patients but also improves their cardiovascular health outcomes. Currently, guidelines and expert consensus documents on lipid management for CHD patients are increasingly abundant, providing critical references for clinical practice. However, most of this evidence focuses on pharmacotherapy (12–14), and in real-world application, suboptimal patient adherence and insufficient treatment intensity often lead to poor rates of lipid control (15–17). Therefore, actively exploring non-pharmacological interventions to manage lipid levels in CHD patients has become an urgent research priority (18, 19).

Non-pharmacological interventions primarily lower lipid levels through weight control, regular exercise, and smoking cessation (20). Relevant guidelines particularly emphasize (21) that comprehensive non-pharmacological interventions should be highly prioritized and recommended in the management of dyslipidemia in patients with CHD to optimize treatment outcomes. However, evidence regarding non-pharmacological interventions for individuals with both CHD and dyslipidemia remains fragmented and lacks systematic, standardized evidence-based guidance, which severely hinders the translation of evidence into clinical practice. Although existing guidelines on lipid management provide a theoretical basis for non-pharmacological interventions, their practical application presents the following limitations: (1) They are mostly confined to macro-level descriptions of basic principles and elements, lacking specific lifestyle intervention measures; moreover, intervention protocols in original studies vary widely, with no unified guidance standards. (2) While they offer detailed descriptions of intervention content and outcomes, the applicability of their recommendations is limited by cultural differences and regional variations in disease profiles. (3) Current evidence lacks systematic synthesis and standardized guidance, making it difficult to meet the urgent needs of clinical practice. Summarizing best evidence is a crucial component of evidence-based nursing, as it synthesizes the highest-quality and most relevant research findings to provide clear and actionable insights for clinical decision-making (22). Therefore, this study aims to conduct a graded retrieval and synthesis of evidence based on the “6S” pyramid evidence resource model (23). In this model, each “S” represents a type of evidence, categorized hierarchically into six levels from the highest to the lowest: computerized decision support systems, evidence-based synopses of topics, synopses of systematic reviews, systematic reviews, synopses of original studies, and original studies. By rigorously evaluating and systematically integrating evidence on non-pharmacological interventions for CHD patients with dyslipidemia according to this model, this study seeks to provide a reference for developing scientific, precise, and efficient clinical intervention protocols.

## 2 Materials and methods

### 2.1 Research design

This study adopted a best-evidence summary design. This methodology aims to address specific clinical questions that require urgent evidence-based answers by systematically retrieving and critically appraising existing evidence resources, such as guidelines and systematic reviews, in order to provide efficient and reliable support for rapid decision-making. It differs in both objectives and scope from systematic reviews, which focus on quantitative synthesis of original research, and from scoping reviews, which aim to map the scope and characteristics of an evidence field. To ensure rigor and transparency, the design and reporting of this study strictly followed the PRISMA-ScR guidelines. Although these guidelines are primarily intended for scoping reviews, their core requirements for systematic searching, screening, data extraction, and reporting transparency align closely with the objective of this study: to comprehensively and transparently summarize and present high-quality evidence. The study protocol was registered with the Center for Evidence-Based Nursing at Fudan University (Registration Number: ES20246942). This study is a secondary analysis of the existing literature and is considered exempt from ethical review.

### 2.2 Establishment of problems

Evidence-based questions were constructed following the PIPOST model proposed by the Evidence-Based Nursing Center of Fudan University (24): (1) Population (P): patients with coronary atherosclerotic heart disease; (2) Intervention (I): non-pharmacological interventions aimed at controlling dyslipidemia in patients with coronary heart disease; (3) Evidence users (P): healthcare professionals, patients, and their families; (4) Outcomes (O): lipid-monitoring indicator measurements; (5) Settings (S): hospitals, communities, and households; (6) Types of evidence (T): clinical decisions, best practices, clinical guidelines, systematic reviews, evidence summaries, expert consensus documents, and relevant original studies.

### 2.3 Literature search strategy

Following the top-down retrieval principle of the “6S” evidence model (23), we searched the following evidence-based resources using a combination of subject headings and free-text terms: (1) Clinical decision support systems: BMJ Best Practice and UpToDate; (2) Guideline websites: the Guidelines International Network (GIN), the National Institute for Health and Care Excellence (NICE), the Scottish Intercollegiate Guidelines Network (SIGN), the New Zealand Guidelines Group (NZGG), the National Guideline Clearinghouse (NGC), and Medlive; (3) Professional association websites: the American Heart Association (AHA), the American College of Cardiology (ACC), the European Society of Cardiology (ESC), the International Atherosclerosis Society (IAS), and the National Lipid Association (NLA); (4) Comprehensive databases: China National Knowledge Infrastructure (CNKI), Wanfang Data, China Biology Medicine disc (CBM), PubMed, Web of Science, CINAHL, and Embase. The search period was set from January 2014 to December 2025. Search terms encompassed: Coronary Disease, Coronary Heart Disease, Coronary Atherosclerotic Heart Disease, Ischemic Heart Disease, Myocardial Ischemia, Acute Coronary Syndrome, Myocardial Infarction, Angina Pectoris; Lipids, Cholesterol, Hyperlipidemias, Dyslipidemias, Hypercholesterolemia, Lipidosis, Blood Lipid Management, and Blood Fat Management. The detailed search strategy is provided in Supplementary Material 1.

### 2.4 Literature inclusion and exclusion criteria

Inclusion Criteria: (1) Patients aged ≥18 years with a diagnosis of coronary heart disease and comorbid dyslipidemia; (2) Interventions involving any structured non-pharmacological strategy (e.g., exercise training, dietary modification, comprehensive lifestyle intervention, or patient education) aimed at improving lipid profiles; (3) Reported outcomes including absolute values or changes in key lipid parameters (e.g., LDL-C, TC, TG, HDL-C); (4) Publication types: clinical guidelines, expert consensus statements, systematic reviews, meta-analyses, evidence summaries, randomized controlled trials, non-randomized controlled trials, or prospective cohort studies; (5) Publications in Chinese or English. Exclusion Criteria: (1) Literature for which the full text is unavailable; (2) Literature deemed to be of low methodological quality (for example, RCTs with an overall high risk of bias per Cochrane RoB tool); (3) Duplicate publications.

### 2.5 Literature quality evaluation

Four researchers independently assessed the included guidelines, while two other researchers independently evaluated the quality of expert consensus documents, standards, and systematic reviews. Discrepancies were resolved through discussion or by consultation with a third researcher. All four researchers had received systematic training in evidence-based methods. The criteria for assessing the quality of the literature were as follows: (1) Clinical Decision Support Tools: As the highest-level resources in evidence-based practice, they are authoritative and comprehensive. Evidence directly relevant to the study topic from clinical decision support tools or evidence summaries was included. (2) Guidelines: Evaluated using the Appraisal of Guidelines for Research and Evaluation (AGREE) II tool (25), which consists of 23 items across 6 domains and 2 global rating items. Each item is scored on a scale from 1 to 7 points. The standardized percentage for each domain was calculated as: (Actual Score − Minimum Possible Score)/(Maximum Possible Score − Minimum Possible Score) × 100%. The recommendation levels were categorized into: Grade A (strongly recommended, with standardized scores ≥60% in all 6 domains), Grade B (recommended, with ≥3 domains scoring ≥30% and <60%), and Grade C (not recommended, with ≥3 domains scoring <30%). Guidelines rated as Grade C were excluded. (3) Expert Consensus: Evaluated using the expert consensus evaluation criteria of the Australian JBI Evidence-Based Health Care Center (2016) (26), which includes 6 assessment items. Each assessor independently selected one of the following responses: “Yes,” “No,” “Unclear,” or “Not applicable.” (4) Systematic Reviews and Meta-Analyses: Methodological quality was assessed using the Assessment of Multiple Systematic Reviews (AMSTAR) tool (27). This instrument consists of 11 items: Was an *a priori* design provided? Was there duplicate study selection and data extraction? Was a comprehensive literature search performed? Was the publication status (e.g., grey literature) used as an inclusion criterion to minimize publication bias? Was a list of included and excluded studies provided? Were the characteristics of the included studies (e.g., design, sample size, outcomes) provided? Was the scientific quality of the included studies assessed and documented appropriately? Was the scientific quality of the included studies used appropriately in formulating conclusions? Were appropriate methods used to combine the findings of studies? Was the likelihood of publication bias assessed? Was any potential conflict of interest stated? For each item, two independent assessors selected one of the following responses: “Yes,” “No,” “Unclear,” or “Not Applicable.” (5) Randomized Controlled Trials: The risk of bias was evaluated using the Cochrane Risk of Bias tool for randomized trials, version 2 (RoB 2) (28). The assessment covered seven specific domains: randomization process; deviations from intended interventions; missing outcome data; measurement of the outcome; and selection of the reported result. For each domain, two independent assessors made a judgment of “Low risk,” “High risk,” or “Unclear risk.”

### 2.6 Summary, classification, and recommendation level of evidence

Two researchers independently extracted evidence, collecting information including publication date, study type, source of evidence, topic of evidence, and specific content. All researchers were involved in the translation, extraction, and synthesis of evidence. The principles for evidence synthesis were as follows (29): (1) When evidence contents were consistent, concise, clear, and professional evidence was prioritized. (2) When evidence contents complemented each other, they were merged into logically structured statements. (3) When evidence contents differed, priority was given based on evidence-based, high-quality, authoritative, and recently published evidence. In this study, evidence was graded using the Australian JBI Centre for Evidence-Based Health Care Evidence Grading and Recommendation System (2014 version), which classifies evidence into levels 1 to 5. Subsequently, two evidence-based nursing experts and three cardiovascular field experts were invited to determine the recommendation level (Grade A: strong recommendation or Grade B: weak recommendation) for each piece of evidence based on the principles of validity, feasibility, appropriateness, and clinical significance (30, 31).

## 3 Results

### 3.1 Literature search results

**Figure 1:**
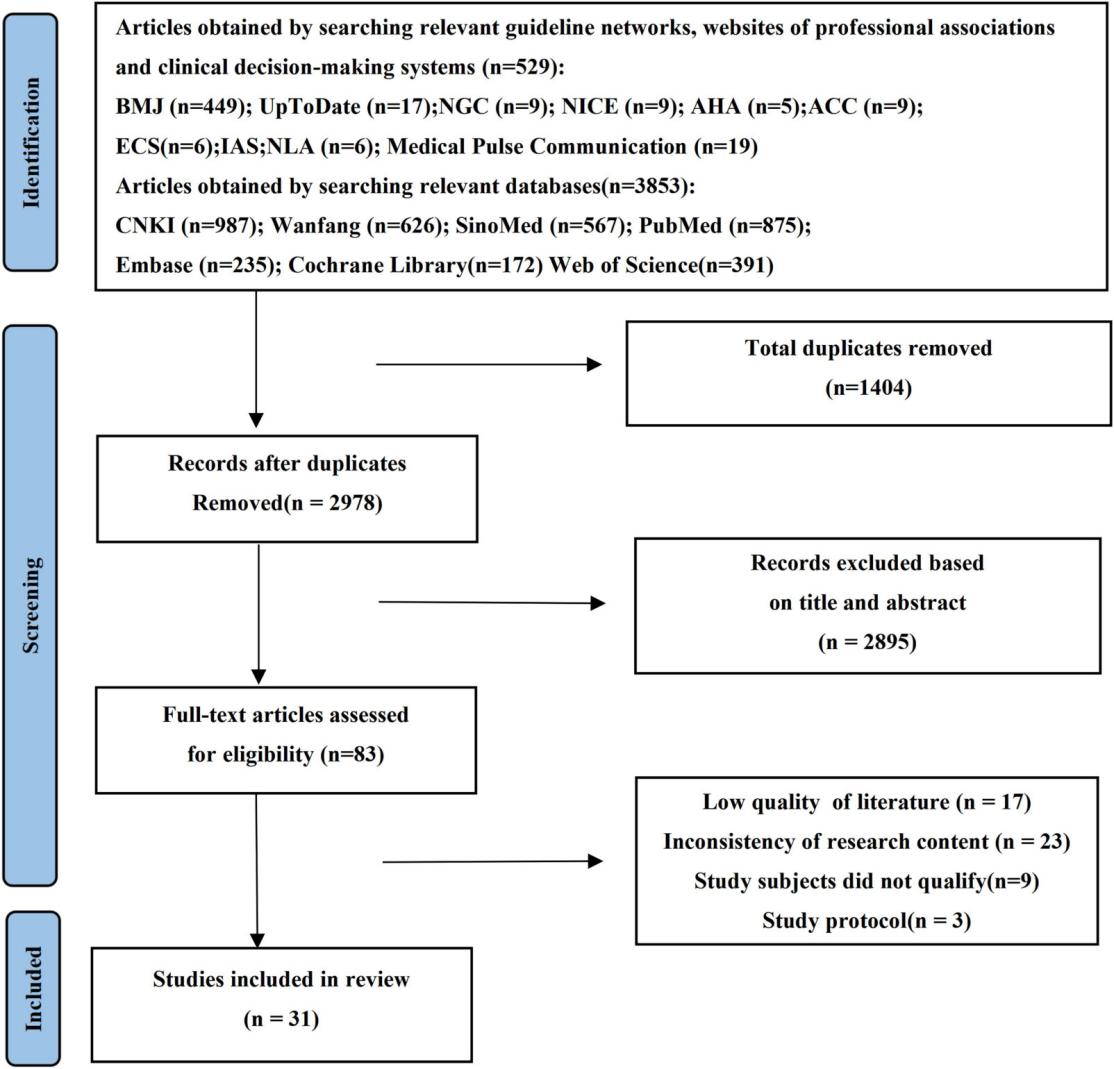
PRISMA flowchart.

A total of 5,212 records were identified through the initial literature search. After screening, a total of 31 studies were ultimately included, comprising 15 English-language publications and 16 Chinese-language publications. The study selection process is presented in Figure 1. The basic characteristics of the included studies are summarized in Table 1.

**Table 1:** Basic characteristics of included studies (*n* = 27).

Author(s)	Year of Publication	Study Type	Source	Topic/Focus
Mach (32)	2025	Guideline	PubMed	Management of Dyslipidemia
Virani (33)	2023	Guideline	PubMed	Management of Chronic Coronary Disease
Kolber (34)	2023	Guideline	PubMed	Prevention and Management of Cardiovascular Disease in Primary Care
Li (35)	2023	Guideline	CNKI	Guideline for Blood Lipid Management
Yan (36)	2022	Guideline	CNKI	Clinical Lipid Testing in China
Pearson (37)	2021	Guideline	PubMed	Management of Dyslipidemia for Cardiovascular Disease Prevention in Adults
Gu (38)	2020	Guideline	CNKI	Healthy Lifestyle for the Prevention of Cardiometabolic Diseases
Hu (39)	2020	Guideline	CNKI	Chinese Guideline for Primary Prevention of Cardiovascular Diseases
Rhee (40)	2019	Guideline	PubMed	Management of Dyslipidemia
Arnett (41)	2019	Guideline	PubMed	Guideline on the Primary Prevention of Cardiovascular Disease
Gu (42)	2019	Guideline	CNKI	Risk Assessment and Management of Cardiovascular Diseases
Grundy (43)	2018	Guideline	PubMed	Guideline on the Management of Blood Cholesterol
Jellinger (44)	2017	Guideline	PubMed	Management of Dyslipidemia and Prevention of Cardiovascular Disease
Li (45)	2024	Expert Consensus	CNKI	Blood Lipid Management for Community-Dwelling Adults
Liu (46)	2023	Expert Consensus	Wanfang	Medical Nutrition Therapy for Dyslipidemia
Liu (47)	2022	Expert Consensus	CNKI	Management of Dyslipidemia in the Elderly
Wang (48)	2021	Expert Consensus	Wanfang	Assessment, Detection, and Intervention of Common Risk Factors for Cardiovascular and Cerebrovascular Diseases
Hu (49)	2019	Expert Consensus	Wanfang	Cholesterol Education Program: Lipid-Lowering Therapy to Reduce Cardiovascular Events
Sun (50)	2024	Systematic Review	Pubmed	Efficacy of Acupuncture on Glucose and Lipid Metabolism in Patients with Coronary Heart Disease
Cui (51)	2024	Systematic Review	Pubmed	Effect of Acupuncture-Related Therapies on Blood Lipid Levels in Patients with Coronary Heart Disease
Jin (52)	2019	Systematic Review	Pubmed	Telehealth Interventions for Secondary Prevention of Coronary Heart Disease
Yu (53)	2022	Meta-Analysis	CNKI	Effect of Resistance Exercise on Cardiovascular Risk Factors in Middle-Aged and Older Adults
Li (54)	2024	Meta-Analysis	Wanfang	Effect of Low-Volume High-Intensity Interval Training on Cardiovascular Disease Risk Factors in Individuals with Obesity or Overweight
Liu (55)	2021	Meta-Analysis	CNKI	Efficacy and Safety of Acupuncture Therapy for Hyperlipidemia
Zhang (56)	2020	Meta-Analysis	CNKI	Efficacy and Safety of Traditional Chinese Exercise Therapy for Stable Angina Pectoris in Coronary Heart Disease
Xu (57)	2020	Meta-Analysis	Pubmed	Effect of Green Tea Consumption on Blood Lipids
Chen (58)	2017	Meta-Analysis	Pubmed	Effectiveness of Endurance Exercise Training in Patients with Coronary Artery Disease
Wu (59)	2017	Meta-Analysis	CNKI	Effect of Lifestyle Intervention on Lipid Profiles in Patients with Dyslipidemia
Liu (60)	2022	Randomized Controlled Trial	CNKI	A Randomized Controlled Trial of Acupoint Catgut Embedding Combined with Atorvastatin Calcium for Hyperlipidemia in the Elderly
Chen (61)	2019	Randomized Controlled Trial	Pubmed	Effect of Smoking and Smoking Cessation on High-Density Lipoprotein Function in Coronary Artery Disease
Zhang (62)	2017	Randomized Controlled Trial	Pubmed	Effects of a Nurse-Led Transitional Care Program on Clinical Outcomes, Health-Related Knowledge, and Physical and Mental Health Status in Chinese Patients with Coronary Artery Disease

### 3.2 Quality evaluation results of the included literature

#### 3.2.1 Quality evaluation results of the guideline

This study included a total of 9 guidelines. The standardized scores and evaluation results for each guideline domain are presented in Table 2.

**Table 2:** Results of guideline quality evaluation.

Included literature	Literature sources	Percentage of standardization by domain (100%)						≥60% of the number of fields (*N*)	≤30% of the number of fields (*N*)	Recommended level
		Scope and Purpose	Participants	Rigour of formulation	Clarity of presentation	Usefulness of the guide	Editorial independence			
Mach (32)	Pubmed	83.3	66.7	81.3	83.3	79.2	91.7	6	0	A
Virani (33)	Pubmed	100.0	88.9	87.8	97.2	37.5	95.8	6	0	A
Kolber (34)	Pubmed	95.9	90.3	88.4	98.6	85.7	95.5	6	0	A
Li (35)	CNKI	77.8	61.1	66.7	66.7	62.5	58.3	5	0	A
Yan (36)	CNIK	88.9	61.1	75.0	83.3	70.8	91.7	6	0	A
Pearson (37)	Pubmed	83.3	61.1	81.3	77.8	75.0	91.7	6	0	A
Gu (38)	CNKI	94.4	55.6	72.9	83.3	66.7	91.7	5	0	A
Hu (39)	CNKI	88.9	55.6	85.4	100	79.2	91.7	5	0	A
Rhee (40)	Pubmed	88.9	77.8	83.3	94.4	83.3	58.3	5	0	A
Arnett (41)	Pubmed	95.4	88.7	98.5	90.3	82.8	90.5	6	0	A
Gu (42)	CNKI	88.9	77.8	79.2	88.9	75.0	83.3	6	0	A
Grundy (43)	Pubmed	95.4	90.7	98.2	92.6	85.6	88.3	6	0	A
Jellinger (44)	Pubmed	90.5	85.6	88.9	92.4	80.7	85.8	6	0	A

#### 3.2.2 Quality evaluation results of the expert consensus

This study included a total of 5 expert consensus documents. The results of the quality assessment are presented in Table 3.

**Table 3:** Results of expert consensus quality evaluation.

Criterion	Li (45)	Liu (46)	Liu (47)	Wang (48)	Hu (49)
Are the sources of viewpoints clearly indicated?	+	+	+	+	+
Do the viewpoints originate from influential experts in the field?	+	+	+	+	+
Are the proposed viewpoints centered on the interests of the relevant study population?	+	+	+	+	+
Are the stated conclusions based on the results of analysis?	+	+	+	+	+
Is existing literature referenced?	+	+	+	+	+
Are there inconsistencies between the proposed viewpoints and previous literature?	?	+	?	+	?

Remark: the green“+” indicates “Yes”, and the yellow “?” indicates “Unclear”.

#### 3.2.3 Quality assessment results of systematic reviews and meta-analyses

This study included 3 systematic reviews and 7 meta-analyses. The quality assessment results are presented in Table 4.

**Table 4:** Results of systematic review and meta-analysis quality evaluation .

Criterion	Sun (50)	Cui (51)	Jin (52)	Yu (53)	Li (54)	Liu (55)	Zhang (56)	Xu (57)	Chen (58)	Wu (59)
Whether the evidence-based questions raised are clear and unambiguous	+	+	+	+	+	+	+	+	+	+
Whether the inclusion criteria were appropriate for this evidence-based question	+	+	+	+	+	+	+	+	+	+
Whether the search strategy is appropriate	+	+	+	+	+	+	+	+	+	+
Whether the database or resources for searching the literature are adequate	+	+	+	+	+	+	+	+	+	+
Whether the literature quality evaluation criteria used are appropriate	+	+	+	+	+	+	+	+	+	+
whether two or more reviewers independently completed the literature quality evaluation	+	+	+	+	+	?	+	+	?	?
Whether certain measures were taken to reduce errors when extracting data	?	?	+	?	+	?	?	?	?	?
whether the method of pooling studies is appropriate	+	+	+	+	+	+	+	+	+	+
Whether the likelihood of publication bias was assessed	+	+	+	+	+	+	+	+	+	+
Whether the recommendations for policy or practice are based on the results of a systematic review	+	+	+	+	+	+	+	+	+	+
whether the proposed further research direction is appropriate	+	+	+	+	+	+	+	+	+	+

Remark: the green“+” indicates “Yes”, and the yellow “?” indicates “Unclear”.

#### 3.2.4 Quality evaluation results of randomized controlled trials

This study included a total of 3 randomized controlled trials. The results of the quality assessment are presented in Table 5.

**Table 5:** Results of randomized controlled trial quality evaluation.

Criterion	Liu (60)	Chen (61)	Zhang (62)
Randomization process	+	+	+
Deviations from intended interventions	−	+	+
Missing outcome data	+	+	+
Measurement of the outcome	+	+	+
Selection of the reported result	+	+	+

Remark: the green“+” indicates “Low risk of bias”.

### 3.3 Evidence description and summary

Based on the principle of evidence synthesis, researchers summarized the evidence. A total of 31 pieces of evidence were summarized, covering seven evidence themes: establishment of a multidisciplinary team, assessment, lipid-lowering targets, lifestyle modification, traditional Chinese medicine non-pharmacological interventions, health education, and follow-up management as shown in Table 6.

**Table 6:** Summary of evidence for non-pharmacological interventions of dyslipidemia in patients with coronary heart disease.

Evidence Theme	Evidence Content	Level of Evidence	Strength of Recommendation
1. Establishing a Multidisciplinary Team	1. A multidisciplinary team including nutrition professionals should be established to collaboratively manage lipid levels in patients with coronary heart disease (35).	Level 5	A
2. Assessment	2. Conduct a comprehensive cardiovascular risk assessment for patients with coronary heart disease as the basis for decision-making on lipid intervention. Develop individualized lipid intervention strategies according to the level of risk (34, 39, 42, 48, 49).	Level 1	A
	3. Low-density lipoprotein cholesterol (LDL-C) is recommended as the primary target. For patients with concurrent diabetes, metabolic syndrome, obesity, or high triglycerides, non-HDL-C should be used as the primary target instead (39).	Level 1	A
3. Lipid-Lowering Targets	4. LDL-C is the primary target, with the following goals (44): (1) Very high-risk patients: LDL-C <1.4 mmol/L (55 mg/dL), or a reduction >50% from baseline; (2) Extremely high-risk patients: LDL-C <1.8 mmol/L (70 mg/dL), or a reduction >50% from baseline; (3) High-risk patients: LDL-C <2.6 mmol/L (100 mg/dL); (4) Moderate- and low-risk patients: LDL-C <3.4 mmol/L (130 mg/dL). Non-HDL-C is a secondary target, with goals approximately 0.8 mmol/L (30 mg/dL) higher than the LDL-C target for the same risk category (32, 43, 49).	Level 2	B
4. Lifestyle Modification			
4.1 Dietary Management	5. Control total caloric intake while meeting daily essential nutritional needs, and appropriately select the proportion of various nutritional components (35).	Level 2	A
	6. Increase intake of fresh fruits and vegetables, whole grains, dietary fiber, and healthy proteins (low-fat dairy, legumes, skinless poultry, fish/seafood, nuts) (32, 34–39, 43).	Level 1	A
	7. Limit total daily fat intake to 20−25 g, replace saturated fatty acids (animal fat, palm oil, etc.) with unsaturated fatty acids (fish, rapeseed oil, olive oil, etc.), and avoid trans fatty acids (hydrogenated vegetable oils, etc.) (32, 37–40, 42, 43).	Level 2	A
	8. Limit dietary cholesterol intake (<300 mg/day) (39, 40, 43).	Level 2	A
	9. Limit intake of processed foods, red meat, sugar-sweetened beverages, and refined carbohydrates (40, 43).	Level 1	A
	10. Limit alcohol intake (men ≤25 g/d, women ≤15 g/d) (38, 40).	Level 1	A
	11. Long-term tea consumption helps lower blood lipids. Moderate consumption of green tea is recommended, with a monthly tea leaf consumption of 50−250 g. However, strong tea is not advised, and tea should not replace all drinking water (38, 57).	Level 5	A
	12. Intake of plant sterols, fish oil, and red yeast rice may help lower blood lipid levels (36).	Level 5	B
4.2 Exercise Management	13. Moderate physical activity is beneficial for regulating lipid levels. Diversified exercise is recommended, and participation in group activities is encouraged (34, 53, 54, 56, 58).	Level 1	A
	14. Patients with coronary heart disease are recommended to perform aerobic exercise 3–4 times per week, for 40 min each session, at moderate to vigorous intensity (40, 54).	Level 1	A
	15. Examples of moderate-intensity aerobic exercise: walking, dancing, household chores, yoga, and Tai Chi. Examples of high-intensity aerobic exercise: brisk walking or running, jumping rope, swimming, basketball, soccer, hiking with load, etc. (38).	Level 1	A
	16. A combination of the two intensities is permissible. Conversion: 1 min of high-intensity aerobic activity is equivalent to 2 min of moderate-intensity aerobic activity (38).	Level 2	B
	17. Elderly patients who cannot meet exercise targets should still be as physically active as their condition allows and avoid sedentary behavior (32, 43).	Level 3	B
4.3 Tobacco Management	18. Avoid all forms of tobacco consumption and avoid exposure to secondhand smoke (32, 43).	Level 1	A
	19. Emphasize the importance of smoking cessation repeatedly to smokers and assist them in quitting. However, electronic cigarettes are not recommended as first-line smoking cessation therapy (43, 56, 57).	Level 1	A
4.4 Weight Management	20. Maintain a BMI of 20–25 kg/m^2^, with waist circumference <94 cm (men) and <80 cm (women), to reduce obesity and insulin resistance (35).	Level 1	A
	21. Adjust diet based on individual calorie needs, food preferences, and medical conditions to avoid weight gain. Overweight and obese individuals should limit staple food intake and control total calories (32).	Level 3	B
	22. Even for individuals who are not overweight, at least 30 min of moderate-intensity physical activity per day is recommended (32, 33, 53).	Level 1	A
	23. Assist overweight/obese patients with coronary heart disease to lose weight in order to reduce cholesterol levels. Pharmacological or surgical intervention may be considered if necessary (33, 35, 45).	Level 1	A
4.5 Sleep and Psychological Management	24. Maintain high-quality sleep for 6–8 h, avoiding excessively short or long sleep duration (37, 38).	Level 1	A
	25. Mental stress can increase serum free fatty acids, which is detrimental to lipid control. It can be managed through psychotherapy, meditation, yoga, etc. (37).	Level 1	A
	26. Patients with coronary heart disease who have psychological disorders such as depression, anxiety, or sleep disorders are advised to seek active treatment (33).	Level 1	A
5. Non-Pharmacological Interventions in Traditional Chinese Medicine (TCM)	27. Acupuncture therapy (50, 51, 55) and acupoint catgut embedding (60) can help regulate blood lipids.	Level 1	A
	28. TCM exercise therapies such as Tai Chi and Baduanjin also aid in lipid control (56).	Level 1	A
6. Health Education	29. Disseminate knowledge about coronary heart disease and lipid control, emphasize the importance of adhering to a healthy lifestyle, and support patients in making beneficial behavioral changes (40, 59).	Level 1	A
7. Follow-up Management	30. Regular lipid testing is crucial for lipid management. For patients on non-pharmacological therapy, lipid levels should be rechecked within the initial 3–6 months. If targets are met, continue non-pharmacological therapy with follow-up tests every 6–12 months. For those consistently meeting targets long-term, annual testing is sufficient (45, 47).	Level 1	A
	31. Provide regular follow-up guidance to patients with coronary heart disease. Follow-up methods include home visits, telephone calls, and telehealth (52, 62).	Level 1	A

## 4 Discussion

### 4.1 Setting lipid management targets based on cardiovascular risk levels is scientifically sound

Evidence summaries 1–3 outline the assessment and lipid-lowering targets for patients with CHD. These targets are closely related to the patient's cardiovascular risk level, with stricter LDL-C control goals required for individuals at higher risk (7). This indicates that applying “normal” reference ranges intended for the general population to CHD patients may fail to achieve adequate risk control. Therefore, clinical practice recommends setting lipid-lowering targets according to the patient's specific risk level. Moreover, since the primary goal of lipid management is the prevention and treatment of CHD, and given the pivotal role of LDL-C in the development of atherosclerosis and the progression of CHD (63), current evidence (39) recommends LDL-C as the primary intervention target in lipid testing—a view widely accepted in clinical practice (64). However, it should be noted that individual responses to lipid-lowering therapy vary. Thus, when establishing lipid-lowering goals, the patient's actual clinical situation should be taken into account to ensure both scientific soundness and clinical feasibility. Evidence summary 4 suggests that lipid management for CHD patients requires multidisciplinary collaboration. In the future, healthcare professionals may refer to this evidence summary to form multidisciplinary management teams that include nutrition, nursing, cardiology, and other relevant disciplines (65). By developing scientific, standardized, and individualized lipid management protocols for CHD, along with comprehensive implementation strategies, the effective integration of lipid management into clinical practice can be promoted.

### 4.2 Strengthen non-pharmacological interventions for patients and promote the adoption of a healthy lifestyle

Evidence Recommendations 5–26 suggest that patients with coronary heart disease can achieve a healthy lifestyle transformation through measures such as dietary control, regular exercise, and smoking cessation, thereby improving lipid management outcomes. Existing literature indicates that a reasonable diet and sustained regular exercise can improve body composition, enhance immunity, and prevent or delay disease progression (66, 67). These practices also exert positive effects on the physical and mental health as well as the quality of life of patients with coronary heart disease (68, 69). The recommendations presented in this study integrate multiple guidelines and are largely consistent with the healthy lifestyles currently advocated (70, 71). However, in real-world clinical practice, to ensure long-term patient benefit, the development of specific plans should fully take into account patient preferences, physical condition, and resource availability. Whenever feasible, dietary and exercise guidance should be provided under supervision to enhance patient adherence and long-term persistence. It is worth noting that the currently recommended dietary patterns are primarily the DASH (Dietary Approaches to Stop Hypertension) diet (in the United States) and the Mediterranean diet (in Europe) (72), which may differ from the traditional dietary habits of populations in various countries. Therefore, future work should actively explore lipid-management dietary patterns that align with local patient preferences and eating habits, and should further evaluate the effectiveness of each dietary model. Evidence Recommendations 27–28 summarize non-pharmacological interventions in Traditional Chinese Medicine (TCM). Modalities such as acupuncture and acupoint catgut embedding, as traditional Chinese non-pharmacological therapies, not only offer advantages including demonstrated efficacy, few adverse reactions, and low cost, but also align well with China's national context and patients’ healthcare practices (73). However, owing to differences in ethnicity, healthcare systems, and cultural backgrounds, the effectiveness and applicability of these non-pharmacological interventions in other countries remain uncertain and require further validation through subsequent research.

### 4.3 Strengthen health education and follow-up management to improve lipid management outcomes

Evidence-based recommendation No. 29 advocates implementing lipid management-related health education for patients with CHD. Previous studies have shown (74) that the treatment rate and control attainment rate for dyslipidemia among CHD patients are 14.1% and 26.6%, respectively. This indicates that improving patients’ knowledge of lipid management and treatment adherence can help enhance the achievement of lipid control targets (75). Regarding implementation approaches, on one hand, the importance of lipid management should be communicated to patients and their families through methods such as distributing educational brochures, sharing instructional videos, and organizing health lectures (76). This helps increase adherence to lipid-lowering therapy, thereby improving lipid management outcomes (77). On the other hand, information technology can be utilized to establish “Internet+” health education platforms, which overcome the temporal and spatial limitations of traditional health education and allow patients and their families to access lipid management-related health education resources anytime and anywhere (78, 79). Evidence summaries No. 30–31 compile the recommended frequency and methods of follow-up. Continuously monitoring lipid levels in CHD patients aids in preventing and managing CHD and reduces the risk of disease recurrence (80). These recommendations define lipid monitoring intervals based on patients’ lipid control attainment rates. However, in clinical practice, it is also advisable to comprehensively consider patients’ lipid levels, treatment regimens, and medication responses to develop more personalized lipid monitoring plans for CHD patients. Regarding follow-up methods, in addition to traditional models such as telephone calls, text messages, and home visits, internet-based follow-up models not only significantly improve patients’ knowledge awareness, health behaviors, and quality of life but also effectively reduce the workload of healthcare professionals (81). Therefore, it is recommended to further investigate internet-based follow-up models in the future, continue to integrate and standardize the informatized follow-up management platform for CHD patients, and build an informatized follow-up platform that enables real-time information sharing and full-process coverage management, thereby advancing traditional follow-up models toward greater informatization.

### 4.4 Limitations

This evidence summary comprehensively focuses on evidence regarding the non-pharmacological management of dyslipidemia in patients with coronary heart disease. It aims to assist healthcare practitioners in efficiently accessing and understanding the evidence, improving lipid control in patients with coronary heart disease, and enhancing their cardiovascular health outcomes. However, our review has several limitations. First, since the literature search was restricted to publications in Chinese and English, studies in other languages were not included, which may introduce language bias. Second, the diversity of patient populations, cancer types, and clinical practices across different healthcare settings may affect the generalizability of our recommendations. Finally, certain innovative non-pharmacological intervention strategies might not have been incorporated into our evidence-based recommendations because their scientific validity, generalizability, and precise effectiveness have not yet been fully established. This limitation highlights the necessity for continued research and evaluation to support these emerging approaches.

## 5 Summary

This study synthesizes evidence from 31 non-pharmacological interventions for dyslipidemia in patients with coronary heart disease, providing valuable resources for clinical practitioners. The findings presented herein are based on existing research conclusions. However, individual patient circumstances may vary, necessitating personalized lipid management strategies tailored to specific clinical situations. When integrating this evidence into clinical practice, healthcare professionals should leverage their expertise, consider unique clinical contexts, and make informed decisions grounded in the best available evidence.
