# Supplementary material for: Summary of the best evidence for non-pharmacological interventions for dyslipidemia in patients with coronary heart disease
Source: Front Cardiovasc Med. 2026 Mar 11;13:1753853. doi: 10.3389/fcvm.2026.1753853 (PMC13013076; doi:10.3389/fcvm.2026.1753853)
Supplement: Supplementary file 1 [file Table1.docx]

# Supplementary Material 1

**Detailed search strategy of PubMed**

| **Search** | **Query** |
| --- | --- |
| #1 | Search: "Dyslipidemias"[Mesh] OR "Hyperlipidemias"[Mesh] OR "Cholesterol"[Mesh] OR "Triglycerides"[Mesh] OR "Lipoproteins"[Mesh] |
| #2 | Search:(Dyslipoproteinemias[Title/Abstract] OR lipid metabolism disorder*[Title/Abstract] OR dyslipoproteinemia[Title/Abstract] OR lipodystrophy[Title/Abstract] OR lipodystrophia[Title/Abstract] OR hyperlipidemia[Title/Abstract] OR hyperlipemia[Title/Abstract] OR hyperlipaemia[Title/Abstract] OR hypercholesterolemia[Title/Abstract]) |
| #3 | Search: (#1) OR (#2) |
| #4 | Search: "Coronary Disease"[Mesh] |
| #5 | Search: (Coronary Heart Disease[Title/Abstract] OR Heart Disease, Coronary[Title/Abstract] OR Coronary Diseases[Title/Abstract] OR cardiovascular diseases[Title/Abstract]) |
| #6 | Search: (#4) OR (#5) |
| #7 | Search: (Diet Therapy"[Title/Abstract] OR "Exercise Therapy"[Title/Abstract] OR "Life Style"[Title/Abstract] OR "Nursing Care"[Title/Abstract] OR "Patient Education as Topic"[Title/Abstract] OR "Behavior Therapy"[Title/Abstract] OR ischemic heart disease[Title/Abstract] OR myocardial infarction[Title/Abstract] OR acute coronary syndrome[Title/Abstract]) |
| #8 | Search: (non-pharmacological[Title/Abstract] OR nonpharmacological[Title/Abstract] OR non-drug[Title/Abstract] OR lifestyle[Title/Abstract] OR "dietary intervention"[Title/Abstract] OR nutrition[Title/Abstract] OR exercise[Title/Abstract] OR "physical activity"[Title/Abstract] OR "smoking cessation"[Title/Abstract] OR "weight loss"[Title/Abstract] OR "behavioral intervention"[Title/Abstract] OR "patient education"[Title/Abstract] OR "nursing intervention"[Title/Abstract]) |
| #9 | Search: (#7) OR (#8) |
| #11 | Search: (systematic review[Title/Abstract] OR meta-analysis[Title/Abstract] OR "evidence summary"[Title/Abstract] OR "best evidence"[Title/Abstract] OR "clinical guideline"[Title/Abstract] OR "consensus statement"[Title/Abstract]) |
| #10 | Search: (((#3) AND (#6)) AND (#9) AND (#9)) |

**Detailed search strategy of Web of Science**

| **Search** | **Query** |
| --- | --- |
| #1 | TS=(("coronary heart disease" OR CHD OR "coronary artery disease" OR CAD OR "ischemic heart disease" OR "myocardial infarction" OR "acute coronary syndrome" OR ACS OR post-PCI OR "post CABG") AND (patient* OR survivor*)) |
| #2 | TS=(dyslipidemia OR dyslipidaemia OR hyperlipidemia OR hyperlipidaemia OR hypercholesterolemia OR hypertriglyceridemia OR LDL OR "low density lipoprotein" OR HDL OR "high density lipoprotein" OR triglyceride OR lipid) |
| #3 | TS=("non-pharmacological" OR nonpharmacological OR "non-drug" OR lifestyle OR "dietary intervention" OR nutrition OR diet OR exercise OR "physical activity" OR "smoking cessation" OR "weight loss" OR "behavioral intervention" OR "patient education" OR "self-management" OR rehabilitation) |
| #4 | TS=("systematic review" OR meta-analysis OR "meta analysis" OR "evidence summary" OR "best evidence" OR "clinical guideline" OR "practice guideline" OR "consensus statement" OR "evidence based") |
| #5 | #3 AND #2 AND #1 AND #4 |

**Detailed search strategy of Professional Associations**

| **Search** | **Query** |
| --- | --- |
| American Heart Association (AHA) | (dyslipidemia OR hyperlipidemia OR "lipid management") AND ("coronary heart disease" OR "coronary artery disease") AND ("dietary" OR "nutrition" OR exercise OR "physical activity" OR "lifestyle") |
| American College of Cardiology (ACC) | (lipid AND ("secondary prevention" OR "coronary disease")) AND (non-pharmacologic OR lifestyle) |
| European Society of Cardiology (ESC) | dyslipidemia AND "coronary artery disease" AND (lifestyle OR diet OR exercise) |
| National Lipid Association (NLA) | ("nutrition therapy" OR "lifestyle intervention") AND "cardiovascular disease" |
